# Linking high-sensitivity CRP levels to cardiac dysfunction in patients with psoriasis

**DOI:** 10.1016/j.ijcha.2025.101839

**Published:** 2025-11-12

**Authors:** Tobias Schupp, Mohammad Abumayyaleh, Michael Behnes, Ibrahim Akin

**Affiliations:** Section of Invasive Cardiology and Electrophysiology, Department of Internal Medicine I, University Medical Center Mannheim, Medical Faculty Mannheim, Heidelberg University, Germany

**Keywords:** CRP, Psoriasis, HFrEF, Cardiac dysfunction, Cardiogenic shock

Chronic inflammation – reflected by an increase of C-reactive protein (CRP) or high-sensitivity CRP (hsCRP) – was demonstrated to be associated with incident heart failure (HF) [1] and to independently impair the prognosis of patients with HF, myocardial infarction and cardiogenic shock [2] by promoting endothelial dysfunction, accelerating atherosclerosis, and driving adverse cardiac remodeling. Chronic inflammation may not only initiate but also accelerate and complicate cardiovascular disease, making it both a trigger and a sustaining factor of vascular and myocardial injury [3]. Although these associations have been well established particularly in HF, data investigated the relationship between inflammatory markers and cardiovascular alterations in patients with non-cardiovascular disease, such as psoriasis, are scarce.

Within the current issue of the *IJC Heart & Vasculature*, Dons *et al.* investigated the association of hsCRP levels and cardiac function assessed by transthoracic echocardiography in 972 patients with psoriasis prospectively enrolled in the PSOCADIA study [4]. The authors found hsCRP correlated with older age, higher body mass index (BMI) and overall higher burden of cardiovascular risk factors. Furthermore, higher hsCRP levels were associated with myocardial dysfunction (defined by left ventricular ejection fraction (LVEF < 50 %) and/or global longitudinal strain (GLS) < 16 %). Despite multivariable adjustment for cardiovascular risk profiles, a hsCRP concentration > 2 mg/L was linked to a 48 % increased risk of myocardial dysfunction, suggesting hsCRP may serve as important biomarker for the detection of early myocardial dysfunction. The authors should be commended for conducting such a large-scale prospective study, which highlights the important association between inflammation and cardiac dysfunction even in patients with psoriasis, that may share important features with patients with cardiovascular disease [5]. Although a causal relationship between hsCRP levels and impaired systolic function cannot be established due to the observational study design, the findings nevertheless support the value of hsCRP measurement for risk stratification, even in patients without established cardiovascular disease.

Several aspects warrant further consideration. The authors demonstrated that the association between hsCRP and diastolic dysfunction was no longer evident after multivariable adjustment, specifically when additionally adjusted for BMI, which typically serves a key factor for metabolic dysfunction, since the metabolic syndrome itself is difficult to assess in everyday clinical practice based on routinely available data. This may further support the notion that diastolic dysfunction is largely promoted and triggered by cardiovascular and metabolic comorbidities, and specifically highlight the role of systemic inflammation for the development of systolic dysfunction [6]. However, the study design does not allow conclusions to be drawn regarding the temporal association between a single hsCRP measurement and the development of myocardial dysfunction, as ongoing subclinical HF itself may promote or sustain systemic inflammatory activity. In this context, it will be of particular interest to investigate in longitudinal studies at what point systemic inflammation – as indicated by elevated hsCRP levels – leads to myocardial dysfunction as measured by LVEF and GLS.

Although numerous studies have demonstrated a clear association between inflammatory markers and an unfavorable prognosis in cardiovascular disease, pharmacological reduction of these markers has not yet shown a significant impact on clinical outcomes. For instance, treatment with colchicine led to a decrease in CRP and interleukin-6 in 278 patients with acute HF within the COLICA trial, however, colchicine did not result in a greater reduction of N-terminal pro B-type natriuretic peptide levels and recurrent HF episodes as compared to placebo therapy [7]. In this regard, the potential impact of psoriasis therapy on systemic inflammatory activity and subsequent HF development should be taken into account, as biologicals have been less commonly used in patients with lowest hsCRP levels within the present study. In this context, the authors demonstrated that hsCRP remained associated with the presence of cardiac dysfunction, even after adjustment for specific psoriasis therapy. As the association between inflammation and HF is once again highlighted in this patient cohort, the question arises which therapeutic agents might be considered in the future to reduce inflammation. From this perspective, sodium–glucose cotransporter 2 (SGLT2) inhibitors were demonstrated to reduce adipose tissue-mediated inflammation and pro-inflammatory cytokine production [8]. Although SGLT2 inhibitors were likely prescribed infrequently during the study period from 2021 to 2024, they could play a central role in future therapy, as preclinical studies have already demonstrated that even the topical application of SGLT2 inhibitors may improve skin changes in psoriasis [9]. Nevertheless, the available data on this topic remain extremely limited. The role these agents may play in patients with psoriasis in clinical practice, and whether a substance can be implemented broadly for the targeted reduction of inflammation for the first time, remains to be evaluated in further prospective studies.

## Declaration of competing interest

The authors declare that they have no known competing financial interests or personal relationships that could have appeared to influence the work reported in this paper.

## References

[1] Burger P.M., Koudstaal S., Mosterd A., Fiolet A.T.L., Teraa M., van der Meer M.G., Cramer M.J., Visseren F.L.J., Ridker P.M., Dorresteijn J.A.N. (2023). C-reactive protein and risk of incident heart failure in patients with cardiovascular disease. J. Am. Coll. Cardiol..

[2] Schupp T., Thiele H., Rassaf T., Mahabadi A.A., Lehmann R., Eitel I., Skurk C., Clemmensen P., Hennersdorf M., Voigt; I., Linke A., Tigges E., Nordbeck P., Jung C., Lauten P., Feistritzer H.J., Buske M., Pöss J., Ouarrak T., Schneider S., Behnes M., Duerschmied D., Desch S., Freund A., Zeymer U., Akin I. (2025). C-reactive protein levels and outcomes in infarct-related cardiogenic shock: data from the ECLS-SHOCK trial. Eur. Heart J. Acute Cardiovasc. Care.

[3] Zhang P., Mo D., Lin F., Dai H. (2025). Relationship between novel inflammatory markers derived from high-sensitivity C-reactive protein and heart failure: a cross-sectional study. BMC Cardiovasc. Disord..

[4] Dons M., Sengelov M., Skaarup K.G., Johansen N.D., Lassen M.C.H., Bogh-Sorensen S., Borchenius J., Davidovski F.S., Landler N.E., Nissen C.V., Hansen P.R., Weber B.N., Zachariae C., Skov L., Biering-Sorensen T. (2025). High-sensitivity C-reactive protein is associated with altered cardiac structure and function in psoriasis: the PSOCADIA study. IJC Heart Vasc..

[5] Zhang L., Wang Y., Qiu L., Wu J. (2022). Psoriasis and cardiovascular disease risk in European and East Asian populations: evidence from meta-analysis and Mendelian randomization analysis. BMC Med..

[6] Le Roux R., Mokotedi L., Fourie S., Manilall A., Gunter S., Millen A.M.E. (2022). TNF-α inhibitors reduce inflammation-induced concentric remodelling, but not diastolic dysfunction in collagen-induced arthritis. Clin. Exp. Rheumatol..

[7] Pascual-Figal D., Núñez J., Pérez-Martínez M.T., González-Juanatey J.R., Taibo-Urquia M., Llàcer-Iborra P., Delgado J., Villar S., Mirabet S., Aimo A., Riquelme-Pérez A., Anguita-Sánchez M., Martínez-Sellés M., Noguera-Velasco J.A., Ibáñez B., Bayés-Genís A. (2024). Colchicine in acutely decompensated heart failure: the COLICA trial. Eur. Heart J..

[8] Cowie M.R., Fisher M. (2020). SGLT2 inhibitors: mechanisms of cardiovascular benefit beyond glycaemic control. Nat. Rev. Cardiol..

[9] Al Bahadly W.K.Y., Bdioui A., Al-Gazally M., Al-Saedi H., Salah S.H.B., Ramadhan M. (2024). The effect of dapagliflozin ointment on induced psoriasis in an experimental model. J. Med. Life.

